# Progress and trends in publication of oral and video presentations at the society of gynecologic oncology annual meeting from 2006 to 2016

**DOI:** 10.1016/j.gore.2021.100758

**Published:** 2021-04-16

**Authors:** Anthony A. Milki, Joshua G. Cohen, Amandeep Kaur Mann, Daniel S. Kapp, John K. Chan

**Affiliations:** a The George Washington University School of Medicine and Health Sciences, Washington, DC, United States; b Department of Obstetrics and Gynecology, UCLA Medical Center, Los Angeles, CA, United States; c Palo Alto Medical Foundation Research Institute, Palo Alto, CA, United States; d Stanford University School of Medicine, Stanford, CA, United States; e California Pacific Medical Center, San Francisco, CA, United States

## Abstract

Previous research has identified factors associated with publication of plenary presentations at the Society of Gynecologic Oncology annual meeting. However, there are no detailed comprehensive trends analyses on the publications of these oral and video presentations over time. In this analysis of 11 annual meetings, we found an increase in clinical rather than translational science publications over time. There was a greater focus on chemotherapy clinical studies and palliative care medicine, with a corresponding decline in publications on surgery. Furthermore, the time interval from presentation to publication shortened over our study period. The evaluation of trends in research publications can inform clinicians and researchers about the quality, competitiveness, and neglected areas of study from national meeting presentations.

## Highlights

• Over 11 years of SGO annual meetings, chemotherapy clinical trials and palliative care publications increased.
• Translational research and surgery publications decreased over time.
• The interval from presentation at the SGO annual meeting to publication in peer-reviewed journals decreased over time.

## 1 Introduction

Medical conferences evolve over time to adapt to novel research trends in their respective fields. Gynecologic oncology research has changed rapidly in the last two decades, and with it the Society of Gynecologic Oncology (SGO) annual meeting, one of the specialty’s largest conferences, has adjusted accordingly (Stewart and Fader, 2017, Huang et al., 2020). Prior studies have identified several factors associated with the subsequent publication of oral plenary content presented at the SGO annual meetings showing that multicenter, international, and cohort studies and randomized controlled trials (RCT) were more likely to be published in peer-reviewed journals (Cohen et al., 2013, Milki et al., 2020). Furthermore, over 85% of oral plenary session led to peer-reviewed publication and 41% of surgical videos were published or accessible online. However these studies did not include an analysis of the trends associated with publication.

In this current report, we evaluated the trends in publication of SGO oral plenaries and videos from 2006 to 2016. More specifically, we determined the publication trends of these presentations based on the study characteristics and time to publication.

## 2 Methods

Data were obtained from SGO annual meeting program booklets posted online between 2006 and 2016. This permitted several years from presentation date to the start of the present study to allow for the peer-review process. Characteristics of presentations were obtained as previously described (Milki et al., 2020). In brief, we studied: type of plenary session, type of study, sample size, research content, year of presentation, number of institutions, academic status, and United States (US) or international authorship. For video presentations, we additionally evaluated cancer type and type of surgery addressed. The topic addressed by every oral plenary abstract was noted, and the five topics with the highest publication rates – palliative care, immunotherapy, chemotherapy clinical trials, surgery, cancer genetics – were explicitly included in our analysis (remainder categorized as “Other”).

For abstracts that led to publication, the publishing journal was identified using a PubMed search of titles, author names, and key terms. For surgical videos, additional YouTube and Google Video internet searches were conducted to determine publication status. We established three study periods – 2006 to 2009, 2010 to 2013, and 2014 to 2016 – and identified trends in journals and in published abstract topics.

Mean time to publication for each studied covariate subcategory was assessed for all presentations. Only publications corresponding to previously presented SGO plenary session content that were original articles were included. Fisher’s exact test and chi-squared analysis were used for statistical analyses, as were described in an earlier study (Milki et al., 2020).

## 3 Results

We divided our 11-year study of SGO annual meeting presentations into three time periods: 2006 to 2009, 2010 to 2013, and 2014 to 2016 (a mean of 53.2 plenaries were presented each year, ranging from 40 to 66 in a given year). Across these time intervals, the percentage of “main” plenary presentations comprising published research increased from 44.2% to 59.4% to 65.4%, with a concurrent decrease in other plenary sessions such as focused and express (p < 0.001). The proportion of clinical research presentations over the three time periods was 72.6% to 80% to 81.1%, whereas the translational and basic science research was measured at 27.4%, 20%, and 18.9%, though this was not statistically different (p = 0.11) (Table 1).

**Table 1:** Characteristics of subsequently published oral plenaries over time.

Factors	2006–2009 (N = 215)	2010–2013 (N = 160)	2014–2016 (N = 127)	*P* Value
**Content**				0.11
Clinical	156 (72.6%)	128 (80.0%)	103 (81.1%)	
Translational/basic science	59 (27.4%)	32 (20.0%)	24 (18.9%)	
**Topics**				0.02
Palliative care	4 (1.9%)	4 (2.5%)	9 (7.1%)	
Immunotherapy	4 (1.9%)	2 (1.3%)	1 (0.8%)	
Chemotherapy clinical trials	15 (7.0%)	18 (11.3%)	13 (10.2%)	
Surgery	23 (10.7%)	15 (9.4%)	9 (7.1%)	
Cancer genetics	65 (30.2%)	30 (18.8%)	21 (16.5%)	
Other^a^	104 (48.4%)	91 (56.9%)	74 (58.3%)	
**Center Type**				0.92
Single	87 (40.5%)	68 (42.5%)	52 (40.9%)	
Multi-center	128 (59.5%)	92 (57.5%)	75 (59.1%)	
**Study Size**				0.96
≤20 patients	7 (3.3%)	8 (5.0%)	5 (3.9%)	
≤50 patients	23 (10.7%)	16 (10.0%)	12 (9.5%)	
≤100 patients	23 (10.7%)	21 (13.1%)	14 (11.0%)	
>100 patients	114 (53.0%)	82 (51.3%)	73 (57.5%)	
Not Applicable^b^	48 (22.3%)	33 (20.6%)	23 (18.1%)	
**Findings**				0.99
Positive	205 (95.4%)	152 (95.0%)	121 (95.3%)	
Negative	10 (4.7%)	8 (5.0%)	6 (4.7%)	
**University Affiliation of First Author**				0.33
Yes^c^	196 (91.2%)	146 (91.3%)	110 (86.6%)	
No^d^	19 (8.8%)	14 (8.8%)	17 (13.4%)	
**Fellowship**				0.21
Yes-3 years	131 (60.9%)	82 (51.3%)	63 (49.6%)	
Yes-4 Years	44 (20.5%)	42 (26.3%)	37 (29.1%)	
No	40 (18.6%)	36 (22.5%)	27 (21.3%)	
**US Region**^e^				0.23
West	12 (6.3%)	7 (5.0%)	8 (7.1%)	
South	15 (7.8%)	11 (7.8%)	12 (10.7%)	
East	36 (18.8%)	37 (26.2%)	20 (17.9%)	
Midwest	20 (10.4%)	11 (7.8%)	3 (2.7%)	
Other US location, multiple centers	109 (56.8%)	75 (53.2%)	69 (61.6%)	
**US vs. International Affiliation of First Author**				0.92
US	192 (89.3%)	141 (88.1%)	112 (88.2%)	
International	23 (10.7%)	19 (11.9%)	15 (11.8%)	
**Study Type**				0.16
Chart review	54 (25.1%)	37 (23.1%)	24 (18.9%)	
Randomized controlled trial	17 (7.9%)	11 (6.9%)	13 (10.2%)	
Cohort	64 (29.8%)	65 (40.6%)	55 (43.3%)	
Translational research	57 (26.5%)	38 (23.8%)	25 (19.7%)	
Other^f^	23 (10.7%)	9 (5.6%)	10 (7.9%)	
**Plenary Session Type**				<0.001
Main	95 (44.2%)	95 (59.4%)	83 (65.4%)	
Focused	120 (55.8%)	38 (23.8%)	43 (33.9%)	
Express	0 (0.0%)	27 (16.9%)	1 (0.8%)	
**Journals**				0.13
Gynecologic Oncology	81 (37.7%)	68 (42.5%)	59 (46.5%)	
Obstetrics and Gynecology	14 (6.5%)	8 (5.0%)	10 (7.9%)	
Clinical Cancer Research	11 (5.1%)	4 (2.5%)	4 (3.2%)	
Cancer	16 (7.4%)	7 (4.4%)	2 (1.6%)	
Journal of Clinical Oncology	16 (7.4%)	8 (5.0%)	5 (3.9%)	
Int. Journal of Gynecological Cancer	7 (3.3%)	13 (8.1%)	4 (3.2%)	
Other	70 (32.6%)	52 (32.5%)	43 (33.9%)	

Data are in column percent and may not add up to 100% due to rounding.

a Other topics include clinical trials, social determinants of health, access to care and cost effectiveness studies, cancer diagnostic guidelines, diseases secondary to cancer, videos, medications with other purposes, chemotherapy, robotically-assisted surgery, intrauterine devices, radiotherapy, surgical outcomes, staging and lymph node mapping, GTPase inhibitors, physician wellness, HPV, PARP inhibitors, Neo-adjuvant treatment, hormonal therapy, cancer predispositions, and imaging

b Not applicable for indicating study size since the content is basic science.

c Academic centers with medical schools.

d Hospital and private affiliations.

e Out of those with abstracts from within the US (N = 523).

f Other study type includes cohort, survey, *meta*-analysis, and decision analysis model.

With regard to topics addressed by published plenaries, the percentage of cancer genetics research declined from 30.2% to 18.8% to 16.5% over time (p = 0.02). Of note, there was a 3.5-fold increase in publication of symptom management and palliative care research (1.9% to 2.5 to 7.1%) (p = 0.02). Meanwhile, surgery studies decreased from 10.7% to 9.4% to 7.1% (p = 0.02) (Table 1). Other characteristics – including center type, study size, and university affiliation – were not associated with significant changes in publication rates over time.

The six journals that most frequently published SGO plenary presentations are shown in Table 1. *Gynecologic Oncology* was the leading peer-review journal for publishing the oral presentations (41.4%). Over the three time periods, 37.7%, 42.5% and 46.5% of meeting research was published in this journal (p = 0.13), although the rate increase was not significant. The presentations also advanced to publication in other journals – most frequently in *Obstetrics and Gynecology*, *Clinical Cancer Research*, *Cancer*, *Journal of Clinical Oncology*, and *International Journal of Gynecological Cancer* – with decreasing rates of publication over time (p = 0.13).

Of all the videos analyzed, 25%, 32.1%, and 42.9% were presented during the three time periods, respectively (p = 0.1). The publication rate of these videos were 30.4% to 47.8% to 21.7% (data not shown; p = 0.01). Surgical videos were published as films on YouTube or institutional websites, as journal manuscripts, or in both media. However, there was no significant trend in publication format over time (p = 0.31), nor was there a significant finding with regard to cancer and surgery type (Table 2).

**Table 2:** Characteristics of subsequently published surgical videos over time.

Factors	2006–2009 (N = 7)	2010–2013 (N = 10)	2014–2016 (N = 6)	*P* Value
**US vs. International**				**0.09**
US	7 (100.0%)	8 (80.0%)	3 (50.0%)	
International	0 (0.0%)	2 (20.0%)	3 (50.0%)	
**Region of US**				0.12
West	4 (57.1%)	1 (10.0%)	1 (16.7%)	
South	2 (28.6%)	1 (10.0%)	0 (0.0%)	
East	1 (14.3%)	5 (50.0%)	1 (16.7%)	
Midwest	0 (0.0%)	1 (10.0%)	1 (16.7%)	
Outside of US	0 (0.0%)	2 (20.0%)	3 (50.0%)	
**Type of center**				0.06
Academic	7 (100.0%)	10 (100.0%)	4 (66.7%)	
Community	0 (0.0%)	0 (0.0%)	2 (33.3%)	
**Cancer type**				0.52
Ovary	1 (14.3%)	0 (0.0%)	1 (16.7%)	
Uterus	2 (28.6%)	3 (30.0%)	0 (0.0%)	
Cervix	3 (42.9%)	3 (30.0%)	1 (16.7%)	
Endometrium	0 (0.0%)	2 (20.0%)	0 (0.0%)	
Vulvar	0 (0.0%)	0 (0.0%)	1 (16.7%)	
Vaginal	0 (0.0%)	0 (0.0%)	0 (0.0%)	
Fallopian tubes	0 (0.0%)	0 (0.0%)	1 (16.7%)	
Recurrent	1 (14.3%)	2 (200%)	2 (33.3%)	
**Surgery Type**				0.17
Robotic surgery	6 (85.7%)	3 (30.0%)	3 (50.0%)	
Laparoscopic surgery	1 (14.3%)	5 (50.0%)	1 (16.7%)	
Open surgery	0 (0.0%)	2 (20.0%)	2 (33.3%)	
**Resection or reconstruction**				0.82
Resection	6 (85.7%)	3 (30.0%)	3 (50.0%)	
Reconstruction	1 (14.3%)	5 (50.0%)	1 (16.7%)	
Neither	1 (14.3%)	0 (0.0%)	0 (0.0%)	
**Morbidity preventing**				0.28
Nerve-sparing	0 (0.0%)	2 (20.0%)	0 (0.0%)	
Fertility preservation	0 (0.0%)	0 (0.0%)	1 (16.7%)	
None	7 (100.0%)	8 (80.0%)	5 (83.3%)	
**Surgery Field**				0.99
Non-Gynecologic Surgery	1 (14.3%)	1 (10.0%)	0 (0.0%)	
Gynecologic surgery	6 (85.7%)	9 (90.0%)	6 (100.0%)	

Data are in column percent and may not add up to 100% due to rounding.

We analyzed the trends in time to publication for oral plenaries based on various research characteristics. The mean time to publication for oral plenary sessions was 14 months (range: 1–117 months). Single center studies were typically published more rapidly than multicenter studies (15 months vs. 21 months; p < 0.001). Time to publication in peer-reviewed journals was longer for cohort studies (18 months), RCTs (23 months), and translational research (21 months) than it was for chart reviews (14 months) (p < 0.001). Finally, the mean time to publication in each time period was 18, 21, and 16 months (p = 0.03). No difference was found in the mean times to publication between studies with positive (18 months; range: 1–95) versus negative results (23 months; range: 5–117) over the time period assessed (p = 0.40).

## 4 Discussion

This study highlights the directions gynecologic oncology research is trending towards, an objective that has not been extensively studied. Our analysis identified an increase in clinical rather than translational research over the course of 11 years of SGO annual meetings. More specifically, we observed a growing emphasis on chemotherapy and palliative care, with a decline in publications pertaining to surgery. It is possible that the observed reduction in translational research publications at the SGO annual meetings would be offset by increases at other conferences. The increased focus on palliative medicine and symptom management is a finding consistent with those of Dumanovsky et al and Morrison et al, which report that palliative care is a rapidly growing field in the United States (Dumanovsky et al., 2016, Morrison et al., 2005). The growth of palliative medicine may be attributed to its recognition as a board-certified specialty in 2007, and perhaps to the landmark Bakitas et al’s ENABLE II trial, which found that palliative care improves survival and comfort for severely ill patients (Bakitas et al., 2015) .The observed increase in abstracts pertaining to palliative medicine at the SGO Annual Meeting therefore follows national trends.

To our knowledge, this is the first study to examine the time to publication associated with conference research characteristics. Study type was among the factors that displayed significant influence on publication time. Expert guidelines often indicate that RCTs lie above cohort studies and retrospective studies in the hierarchy of evidence (Sackett, 1986, Llewellyn-Bennett et al., 2016). The completion of an RCT is a rigorous, expensive, and time-consuming prospective process, which may explain the additional time needed prior to appearance in a peer-reviewed journal. This study does not demonstrate an association between academic status, US vs. international authorship, or positive vs. negative findings on time elapsed from conference to journal appearance.

This report has several limitations, several of which have been discussed in an earlier publication (Milki et al., 2020). It would be beneficial for subsequent studies to identify publication trends in poster presentations, an analysis that was not included in the present research. This study includes an odd number of years, and time period divisions therefore did not include an equal number of years. Our analysis of published surgical videos involved a sample size of only 23, reducing the significance of our findings. Nonetheless, this is the first study to extensively identify time trends in published research emerging from the SGO annual meeting.

## Informed consent

This study did not involve human subjects and therefore did not require the receipt of informed consent.

## Declaration of Competing Interest

The authors declared the following potential conflict of interest with respect to the research, authorship, and/or publication of this article: John K. Chan is an honoraria speaker/consultant for: AbbVie, Acerta, Aravive, AstraZeneca, Clovis, Eisai, GlaxoSmithKline, Merck, and Roche. None of the other co-authors have conflicts of interest.

## References

[b0005] Bakitas M.A., Tosteson T.D., Li Z. (2015). Early versus delayed initiation of concurrent palliative oncology care: patient outcomes in the ENABLE III randomized controlled trial. J. Clin. Oncol..

[b0010] Cohen J.G., Kiet T., Shin J.Y. (2013). Factors associated with publication of plenary presentations at the Society of Gynecologic Oncologists annual meeting. Gynecol. Oncol..

[b0015] Dumanovsky T., Augustin R., Rogers M. (2016). The growth of palliative care in U.S. hospitals: a status report. J. Palliat. Med..

[b0025] Huang X.Z., Jia H., Xiao Q. (2020). Efficacy and prognostic factors for PARP inhibitors in patients with ovarian cancer. Front. Oncol..

[b0030] Llewellyn-Bennett R., Bowman L., Bulbulia R. (2016). Post-trial follow-up methodology in large randomized controlled trials: a systematic review protocol. Syst Rev..

[b0035] Milki A.A., Cohen J.G., Kaur Mann A., Kapp D.S., Chan J.K. (2020). Publication of oral and video presentations from the Society of Gynecologic Oncology annual meeting over 11 years - What characteristics were important?. Gynecol. Oncol. Rep..

[b0040] Morrison R.S., Maroney-Galin C., Kralovec P.D. (2005). The growth of palliative care programs in United States hospitals. J. Palliat. Med..

[b0045] Sackett D.L. (1986). Rules of evidence and clinical recommendations on the use of antithrombotic agents. Chest.

[b0050] Stewart K.I., Fader A.N. (2017). New developments in minimally invasive gynecologic oncology surgery. Clin. Obstet. Gynecol..

